# Supplementary material for: Harmine and Piperlongumine Revert TRIB2-Mediated Drug Resistance
Source: Cancers (Basel). 2020 Dec 9;12(12):3689. doi: 10.3390/cancers12123689 (PMC7763856; doi:10.3390/cancers12123689)
Supplement: Supplementary file 1 [file cancers-12-03689-s001.zip › cancers-1020898-supple-XML/cancers-1020898-supple-XML.pdf]

Supplementary Materials: Harmine and Piperlongumine Revert TRIB2-Mediated Drug Resistance

Susana Machado, Andreia Silva, Ana Luísa De Sousa-Coelho, Isabel Duarte, Inês Grenho, Bruno Santos, Victor Mayoral-Varo, Diego Megias, Fátima Sánchez-Cabo, Ana Dopazo, Bibiana I. Ferreira and Wolfgang Link

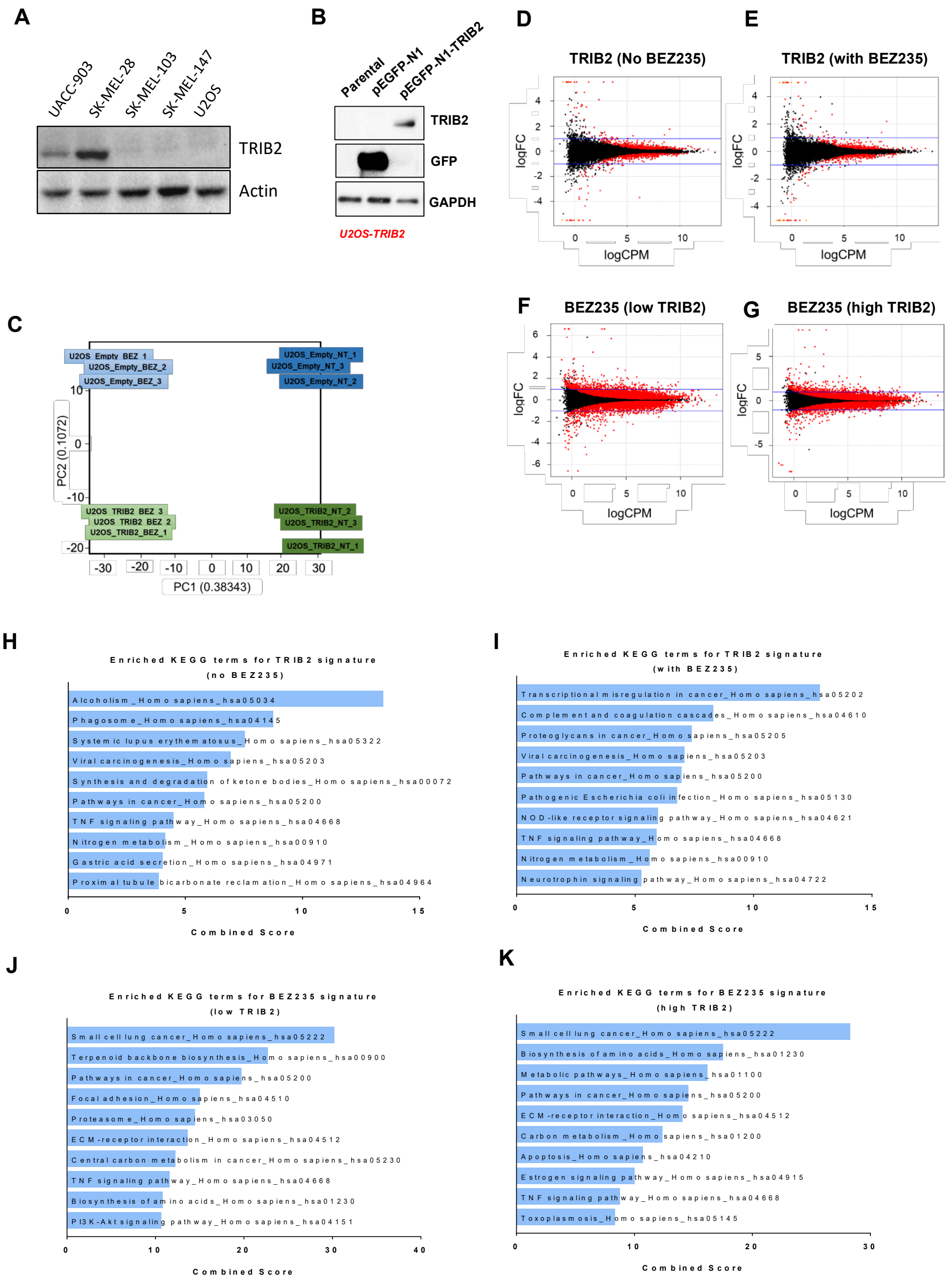

Figure S1. TRIB2 overexpression alters the transcriptional signature in cell lines and affects biological pathways. (**A**) Western-Blot analysis of TRIB2 protein levels in a panel of cell lines. Actin was used as a loading control. (**B**) Western-Blot analysis of TRIB2 protein levels in U2OS (Parental), U2OS-empty (pEGFP-N1) and U2OS-TRIB2 (pEGFP-N1-TRIB2) cell lines. GAPDH was used as a loading control. (**C**) PCA analysis showing the variance produced by BEZ235 treatment and TRIB2 overexpression. BEZ235 treatment produced a stronger effect (greater PCA component), however, both BEZ235 and

TRIB2 have a significant effect on the cells. (**D**–**G**) MA plots show the correlation between read counts (log CPM) and fold change (log2FC). Red dots indicate differentially expressed genes. Horizontal lines indicate fold change threshold (−0.5 and 0.5) to indicate significant fold change. The higher the read count and the higher the fold change, the greater the confidence in the DEGs. D and E display the TRIB2 contribution to gene differential expression on U2OS cells untreated (**D**) and BEZ235-treated cells (**E**), and (**F**) and (**G**) display the BEZ235 treatment contribution to gene differential expression in cells with low (F) and high TRIB2 (G). BEZ235 treatment (**F**–**G**) showed more DEGs than TRIB2 overexpression (**D**–**E**). (**H**–**K**) Top 10 KEGG terms enrichment analysis in TRIB2 signature in untreated (**H**) and BEZ235-treated cells (**I**), and BEZ235 signature in cells with low (**J**) and high TRIB2 (**K**). KEGG term enrichment analysis was performed using the web tool Enrichr by computing the Combined Score, which is obtained by multiplying the log of the *p*-value (the Fisher exact test) by the Z-score. The longer the horizontal bar, the higher the statistical confidence of the term.

A

Drug	Pubchem ID	Enrichment Score	p-value	Mode of Action
Maprotiline	4011	0.9	0.0001	norepinephrine reuptake inhibitor (27)
Cromoglicic acid	2882	0.907	0.01795	chloride channels inhibitor (32)
Arachidonyl trifluoromethane	5280436	0.873	0.03292	PLA_2 inhibitor (33)

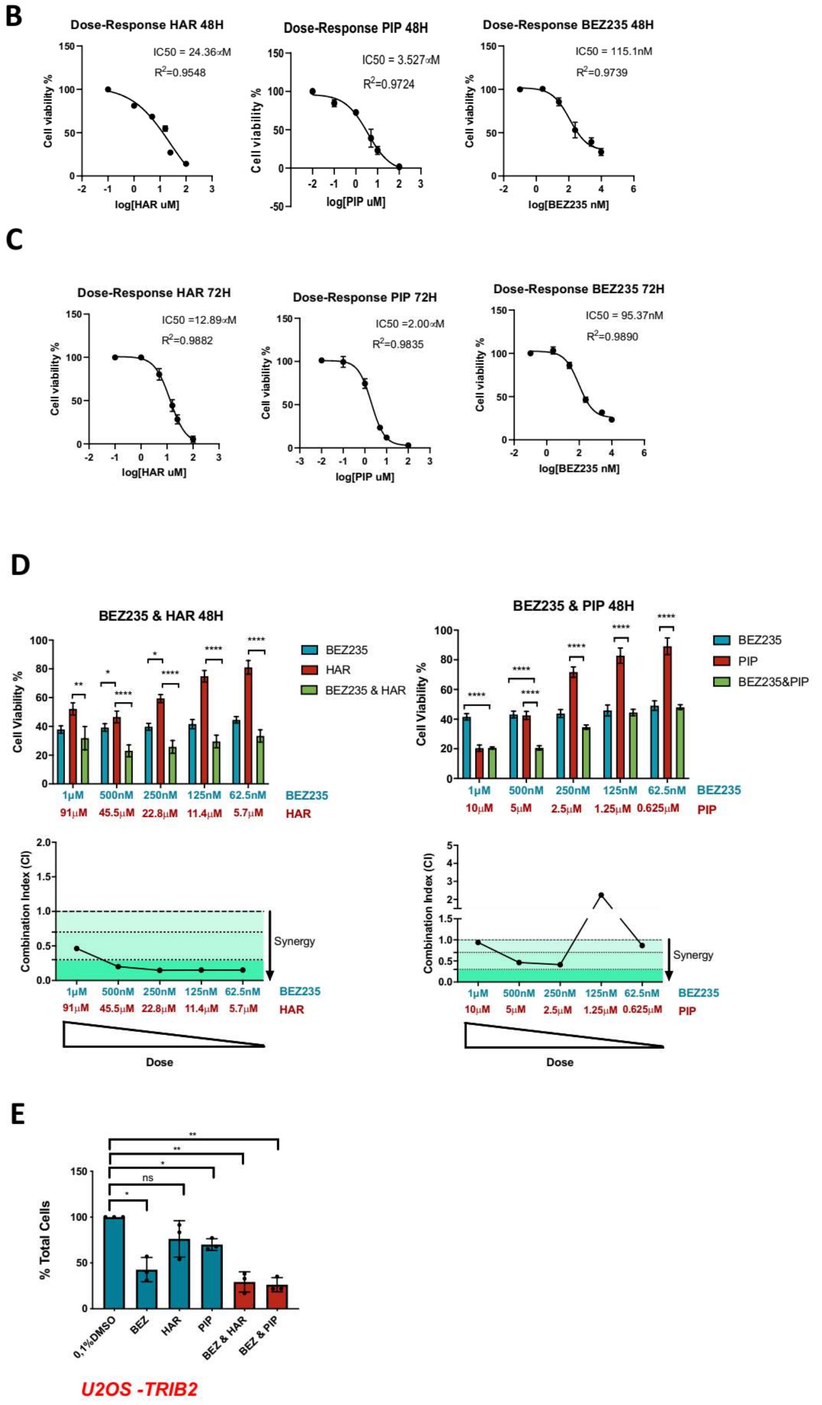

Figure S2. HAR and PIP synergize with BEZ235 to induce cell death (**A**) A cMAP query was performed to identify drugs that generated similar transcriptional signatures of TRIB2 on MCF7, PC3 and MDA-MB-231 cell lines. Drugs were selected according to *p*-value < 0.05 and lower enrichment score. (**B**) and (**C**) represent dose-response curves of HAR, PIP and BEZ235 in U2OS-TRIB2 cell line for 48H and 72H, respectively. Cells were treated with 6 serial dilutions of HAR, PIP or BEZ235 for

the indicated time and cell viability was determined. Results were normalized and fitted with non-linear regression (log inhibitor vs response with variable slope and 4 parameters). Results generated from three independent experiments with triplicates. (**D**) U2OS-TRIB2 cells were treated with BEZ235, HAR or PIP alone, BEZ235 and HAR or BEZ235 and PIP combined, with 48 h treatment. Cell viability was inferred by MTT assay. Synergy was determined by the Chou Talalay method. Combination index (CI) < 1 denotes synergism, CI > 1 antagonism, and CI = 1 additive. BEZ235 and HAR displayed synergy in all concentrations while BEZ235 and PIP displayed synergy in all concentrations except the 125 nM (BEZ235)/1.25 µM (PIP) combination. (**E**) Cell death assay by trypan exclusion assay was performed on U2OS-TRIB2 cells treated with 250 nM BEZ235 and 22.8 µM HAR (the chosen synergic concentration) or 250 nM BEZ235 and 2.5 µM PIP (the chosen synergic concentration) for 72 h. The cell death assay displays a decrease in the total number of cells in each condition, compared to DMSO control. *p*-values were obtained from unpaired *t*-test with Welch correction, (*) $p < 0.1$, (**) $p < 0.01$, (ns) $p > 0.1$ The mean ± SEM from three independent experiments is shown.

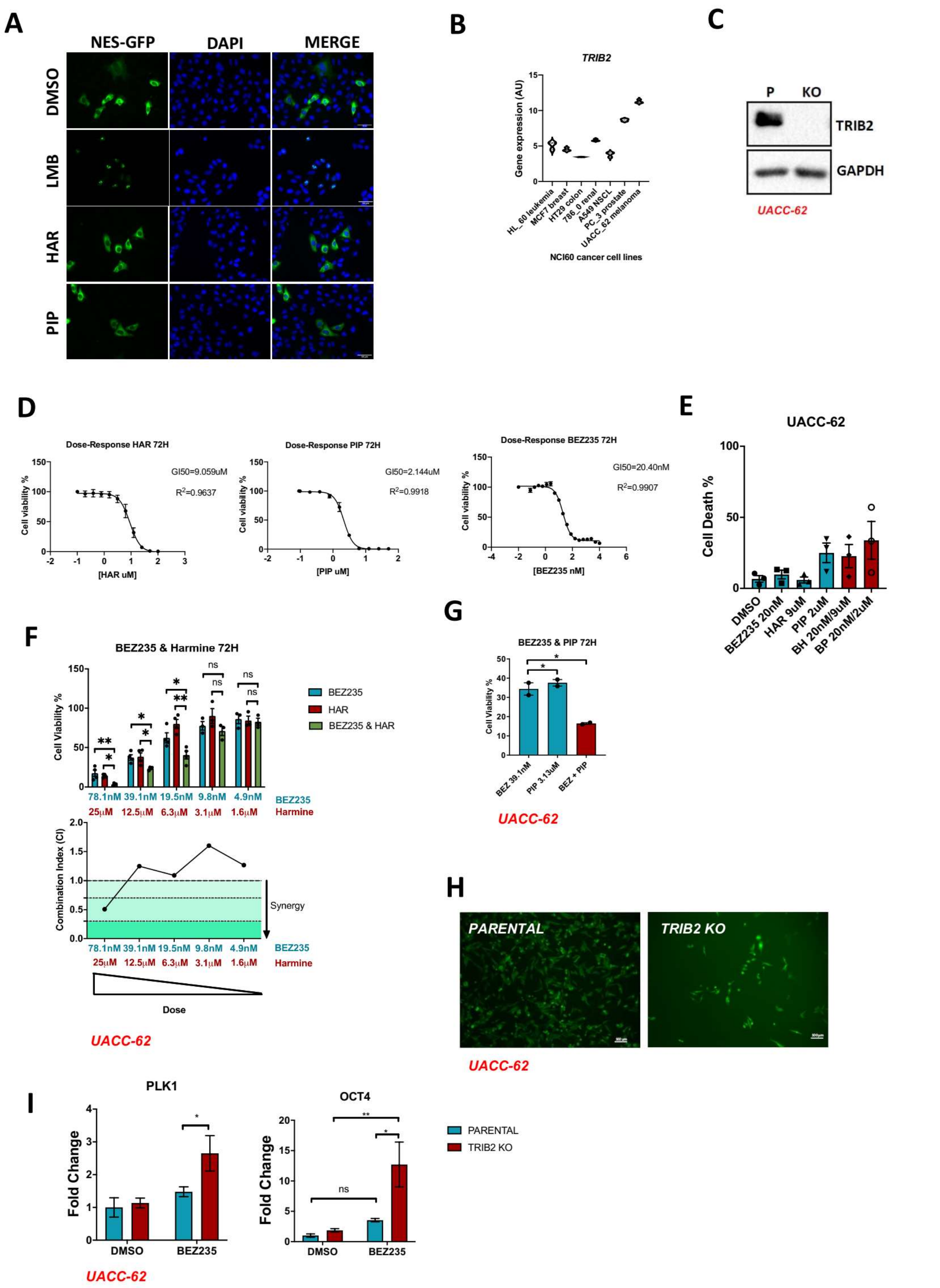

Figure S3. HAR and PIP synergize with BEZ235 to induce FOXO transcriptional activity in UACC-62 cell line (**A**) U2OS-NES-GFP cells were treated with 16 and 13 µM of HAR or PIP, respectively for 1 h. DMSO and LMB were used as negative and positive controls respectively, for FOXO localization. DMSO-treated cells continued to display FOXO mainly in the cytoplasm, while LMB, HAR and PIP-treated cells displayed FOXO mainly in the nucleus. (**B**) Expression analysis of TRIB2 levels in selected cancer cell lines (NCI60 panel, GDS4296) [76]. (**C**) Immunoblot of TRIB2 in UACC-62 cells. GAPDH was used as a loading control. UACC-62 parental cell line (P) was used as a control for TRIB2 KO validation. (**D**) UACC-62 parental cells were treated with serial dilutions of HAR, PIP or BEZ235 for

72 h and cell viability percentage was determined to generate dose-response plots. Results were normalized and fitted with non-linear regression (log inhibitor vs response with variable slope and 4 parameters). (**E**) Cell death assay by trypan exclusion was performed on parental UACC62 cells treated with 20 nM BEZ235 and 9 μM HAR or 20 nM BEZ235 and 2 μM PIP for 72 h. The cell death assay displays an increase of cell death when cells are treated with each drug alone and combined, compared to DMSO control. Statistical significance was determined by 1-Way ANOVA and Tukey multiple comparisons test, with *p*-value < 0.05. Results obtained from 3 independent experiments. (**F**) Parental UACC62 cells were treated with BEZ235, HAR, and BEZ235 and HAR combined, for 72 h. Cell viability was inferred by MTT assay. Statistical significance was determined by 1-Way ANOVA with multiple testing with SIDAK's multiple comparisons test with (*) $p < 0.05$ and (**) $p < 0.01$, (ns) $p > 0.1$. Synergy was determined by the Chou Talalay method. Combination index (CI) <1 denotes synergism, CI > 1 antagonism, and CI = 1 additive. BEZ235 and HAR displayed synergy in all concentrations while BEZ235 and PIP displayed synergy in all concentrations except the 125 nM (BEZ235)/1.25 μM (PIP) combination. (**G**) Parental UACC62 cells were treated with BEZ235, PIP or BEZ235 and PIP combined, with 72 h treatment. Cell viability was inferred by MTT assay. Statistical significance was determined by 1-Way ANOVA with multiple testing with SIDAK's multiple comparisons test with (*) $p < 0.05$. (**H**) Subcellular localization of GFP-FOXO3 stably transfected into parental UACC62 cells and UACC-62 TRIB2 KO cells. (**I**) Parental UACC62 cells were treated with 100 nM BEZ235 or 0.1% DMSO (control) for 72 h. The upregulation of the FOXO target genes PLK and OCT4 was potentiated with TRIB2 KO. Statistical significance was determined by 2-Way ANOVA with multiple testing with SIDAK's and Tukey's multiple comparisons test with (*) $p < 0.05$ and (**) $p < 0.01$, (ns) $p > 0.1$.

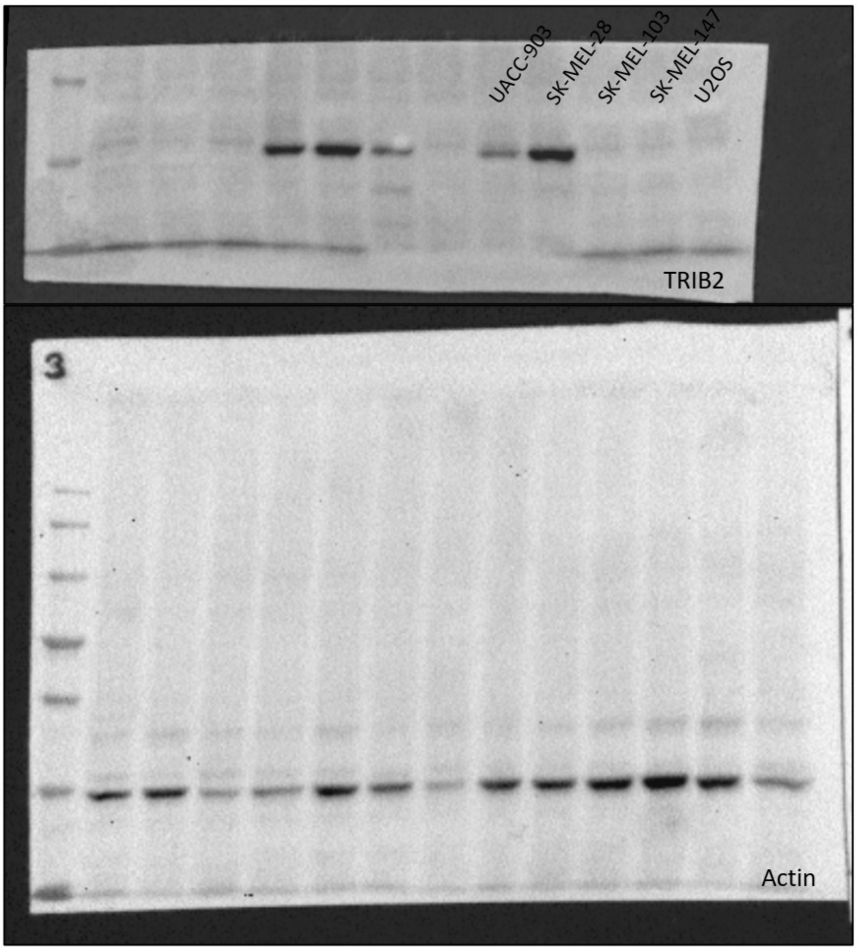

Figure S4. Uncropped Western-Blot analysis of TRIB2 protein levels in a panel of cell lines (refers to Figure S1A). Actin was used as a loading control.

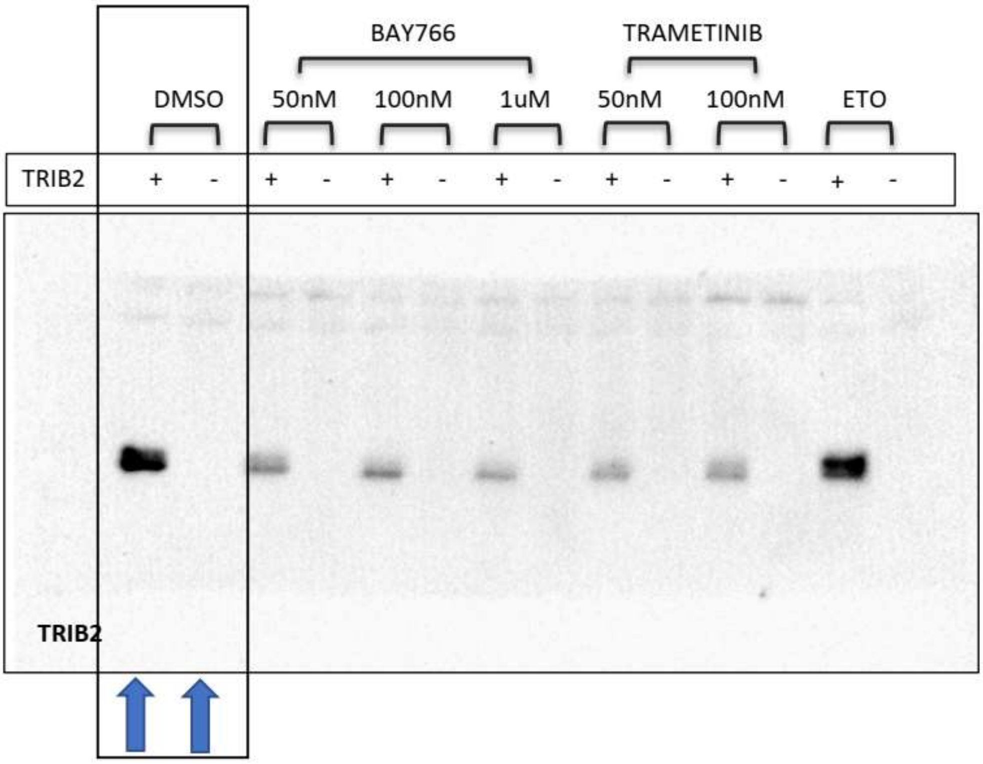

Figure S5. Uncropped immunoblot of TRIB2 in UACC-62 cells for TRIB2 KO validation (refers to Figure S3C). UACC-62 parental cell line (P) was used as a control for TRIB2 KO validation.

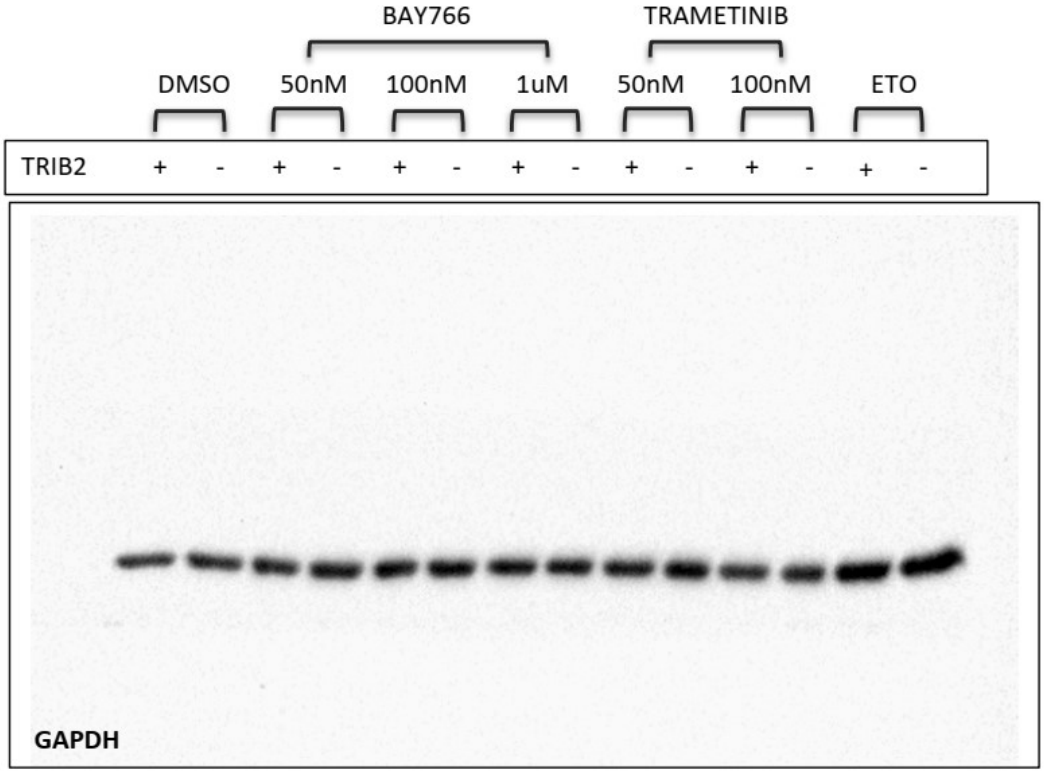

Figure S6. Uncropped immunoblot of GAPDH in UACC-62 cells for TRIB2 KO validation (refers to Figure S3C). GAPDH was used as a loading control. UACC-62 parental cell line (P) was used as a control for TRIB2 KO validation.

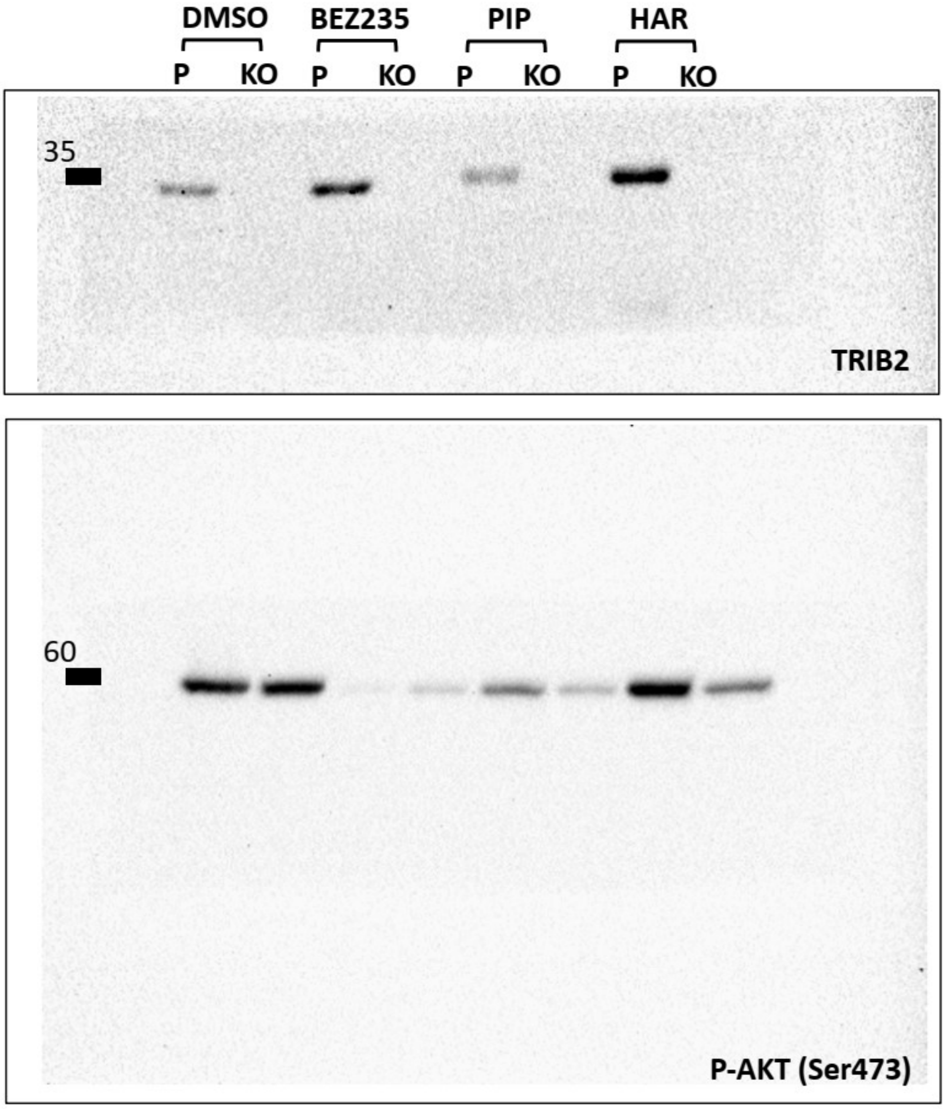

Figure S7. Uncropped immunoblot for p-AKT in parental UACC62 cells (P) and UACC-62 TRIB2 knockout cells (KO), treated with 16 μM HAR or 13 μM PIP for 2 h. Refers to Figure 3F.

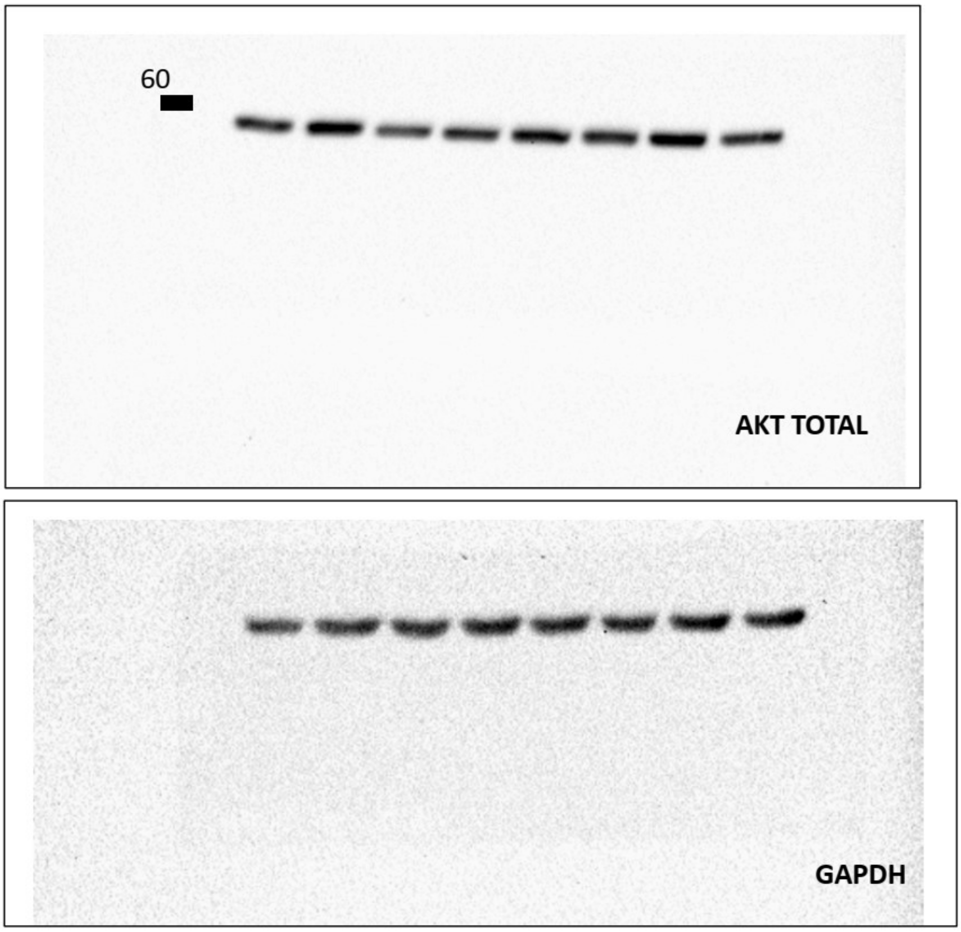

Figure S8. Uncropped immunoblot for GAPDH in parental UACC62 cells (P) and UACC-62 TRIB2 knockout cells (KO), treated with 16 μM HAR or 13 μM PIP for 2 h. Refers to Figure 3F.

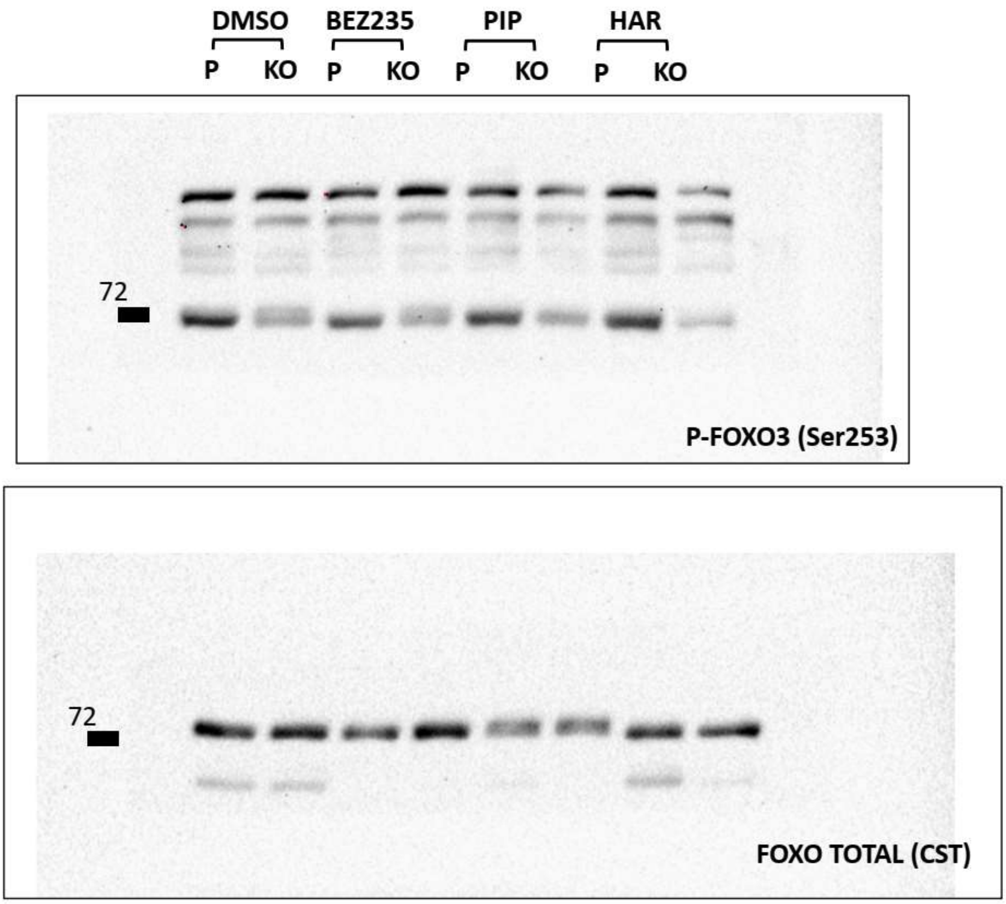

Figure S9. Uncropped immunoblot for p-FOXO3 and FOXO total in parental UACC62 cells (P) and UACC-62 TRIB2 knockout cells (KO), treated with 16 µM HAR or 13 µM PIP for 2 h. Refers to Figure 3F.

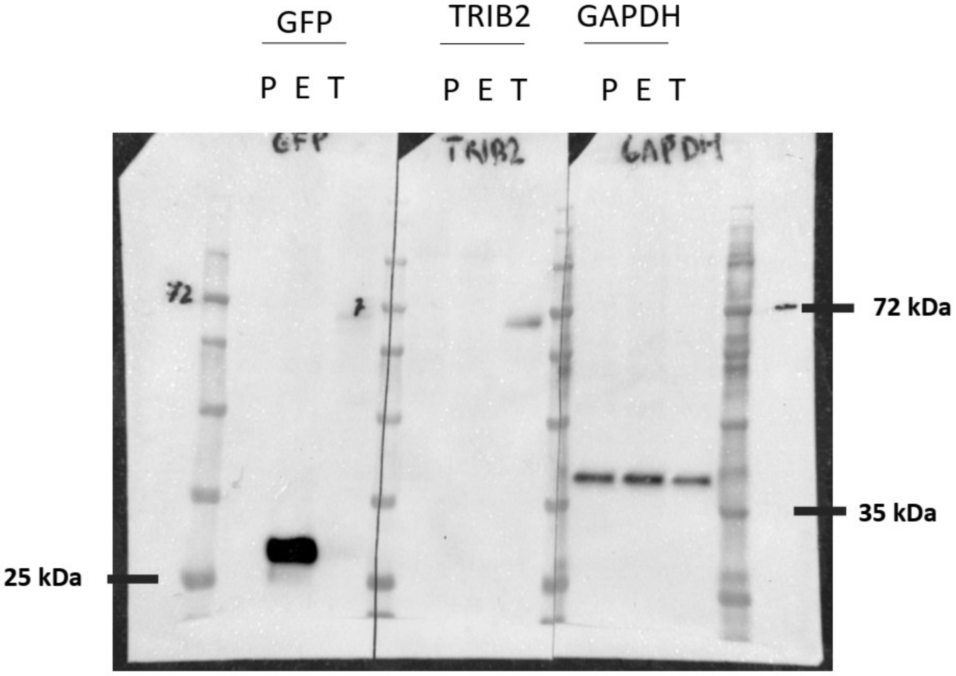

Figure S10. Uncropped Western-Blot analysis of TRIB2 protein levels in U2OS (Parental), U2OS-empty (pEGFP-N1) and U2OS-TRIB2 (pEGFP-N1-TRIB2) cell lines. GAPDH was used as a loading control. Refers to Figure S1B.

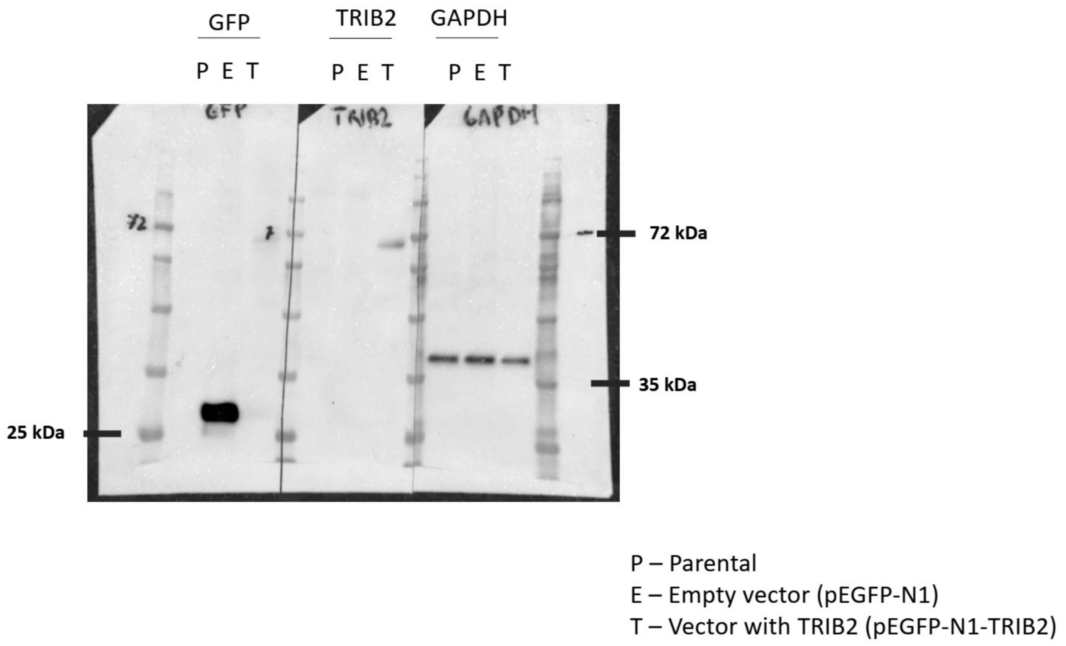

Figure S11. Uncropped Western-Blot analysis of TRIB2 protein levels in U2OS (Parental), U2OS-empty (pEGFP-N1) and U2OS-TRIB2 (pEGFP-N1-TRIB2) cell lines. GAPDH was used as a loading control. Refers to Figure S1B.

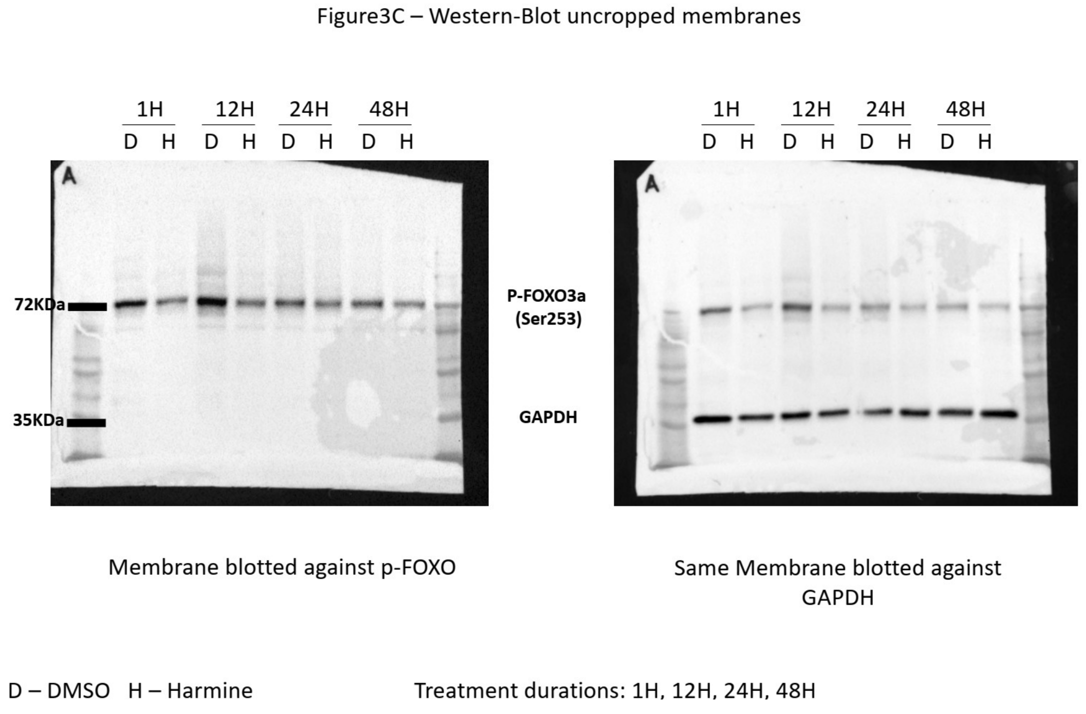

Figure S12. Uncropped Western-Blot for p-FOXO3a and GAPDH in the U2foxRELOC cell line treated with 50 µM HAR (H) for 1, 12, 24 and 48 h. DMSO (**D**) was used as a control for HAR treatment. GAPDH was used as loading control. Refers to Figure 3C.

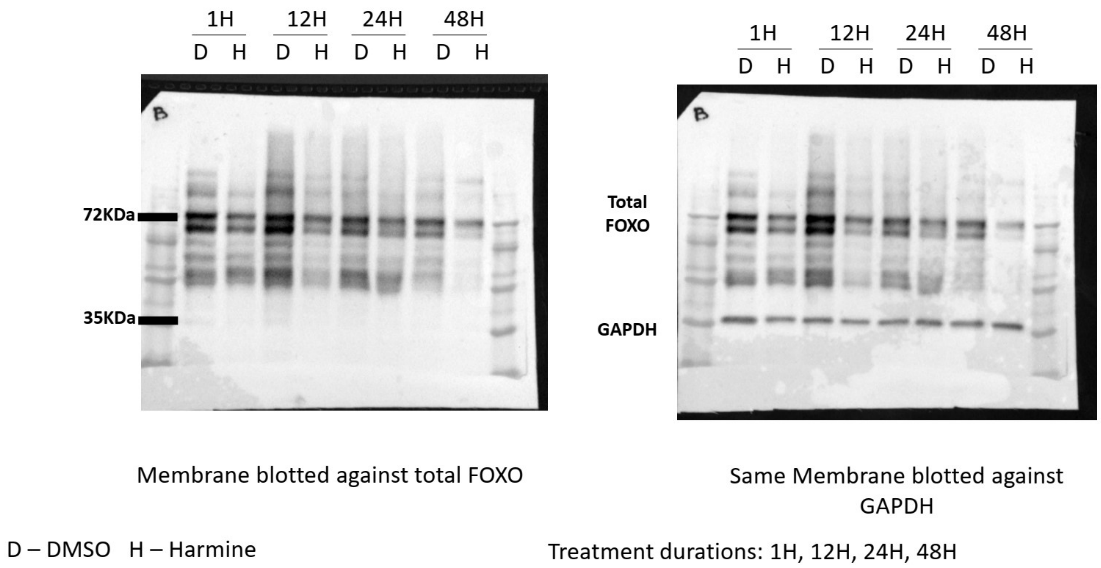

Figure S13. Uncropped Western-Blot for total FOXO and GAPDH in the U2foxRELOC cell line treated with 50 µM HAR (H) for 1, 12, 24 and 48 h. DMSO (**D**) was used as a control for HAR treatment. GAPDH was used as loading control. Refers to Figure 3C.

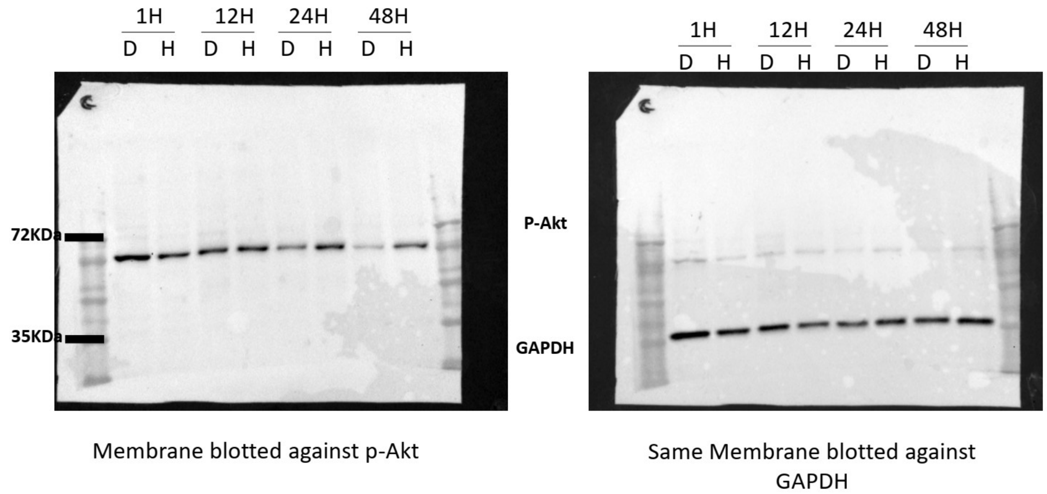

Figure S14. Uncropped Western-Blot for p-Akt and GAPDH in the U2foxRELOC cell line treated with 50 µM HAR (H) for 1, 12, 24 and 48 h. DMSO (**D**) was used as a control for HAR treatment. GAPDH was used as loading control. Refers to Figure 3C.

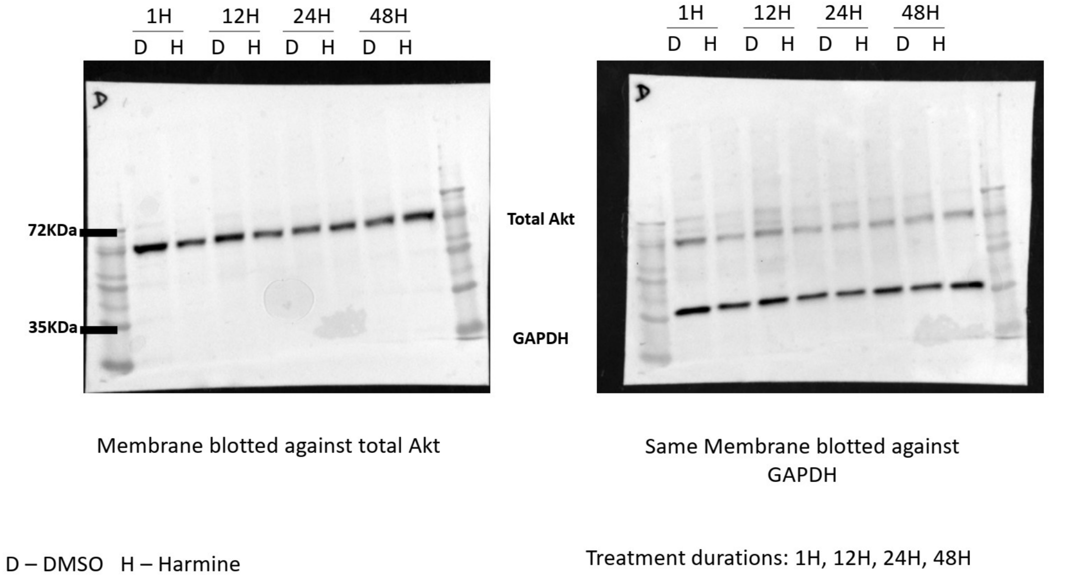

Figure S15. Uncropped Western-Blot for total Akt and GAPDH in the U2foxRELOC cell line treated with 50 µM HAR (H) for 1, 12, 24 and 48 h. DMSO (**D**) was used as a control for HAR treatment. GAPDH was used as loading control. Refers to Figure 3C.

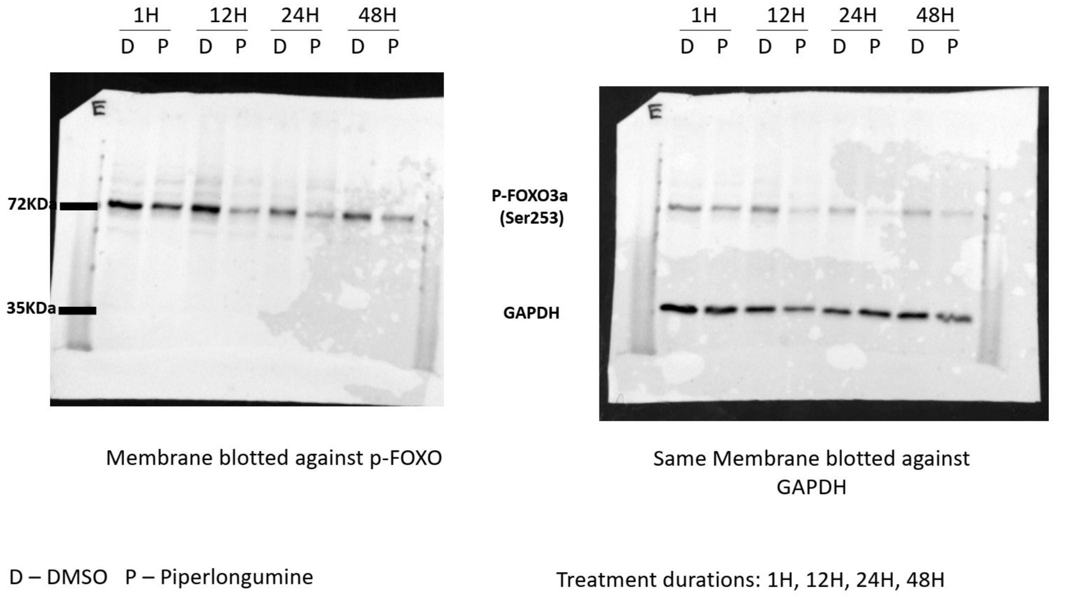

Figure S16. Uncropped Western-Blot for p-FOXO3a and GAPDH in the U2foxRELOC cell line treated with 50 µM PIP (P) for 1, 12, 24 and 48 h. DMSO (**D**) was used as a control for PIP treatment. GAPDH was used as loading control. Refers to Figure 3D.

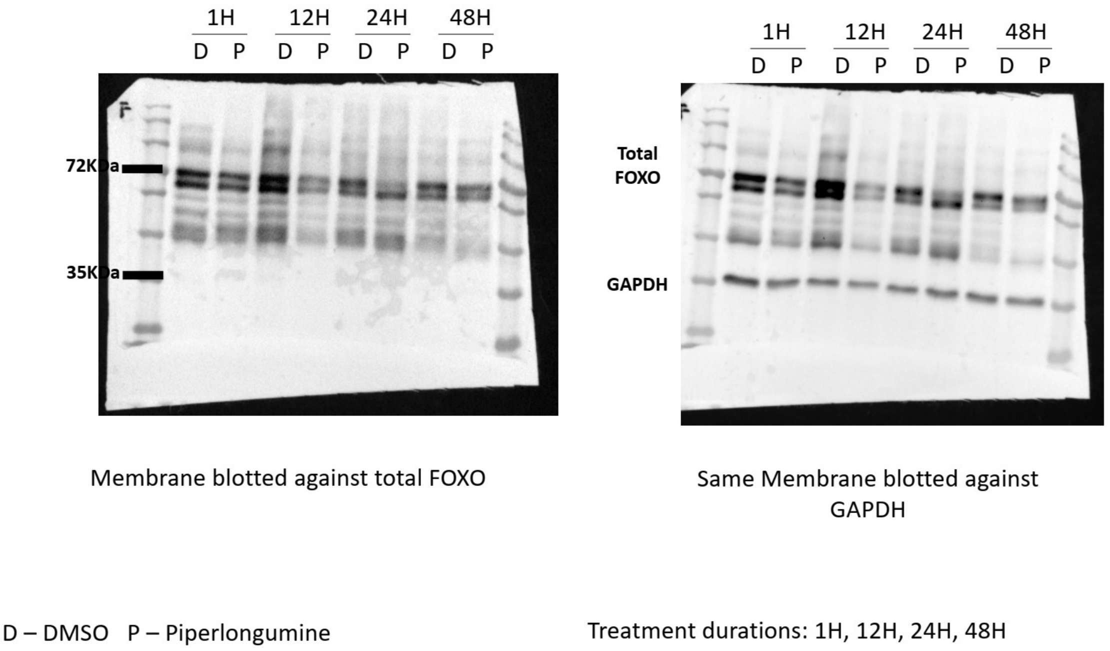

Figure S17. Uncropped Western-Blot for total FOXO and GAPDH in the U2foxRELOC cell line treated with 50 μM PIP (P) for 1, 12, 24 and 48 h. DMSO (**D**) was used as a control for PIP treatment. GAPDH was used as loading control. Refers to Figure 3D.

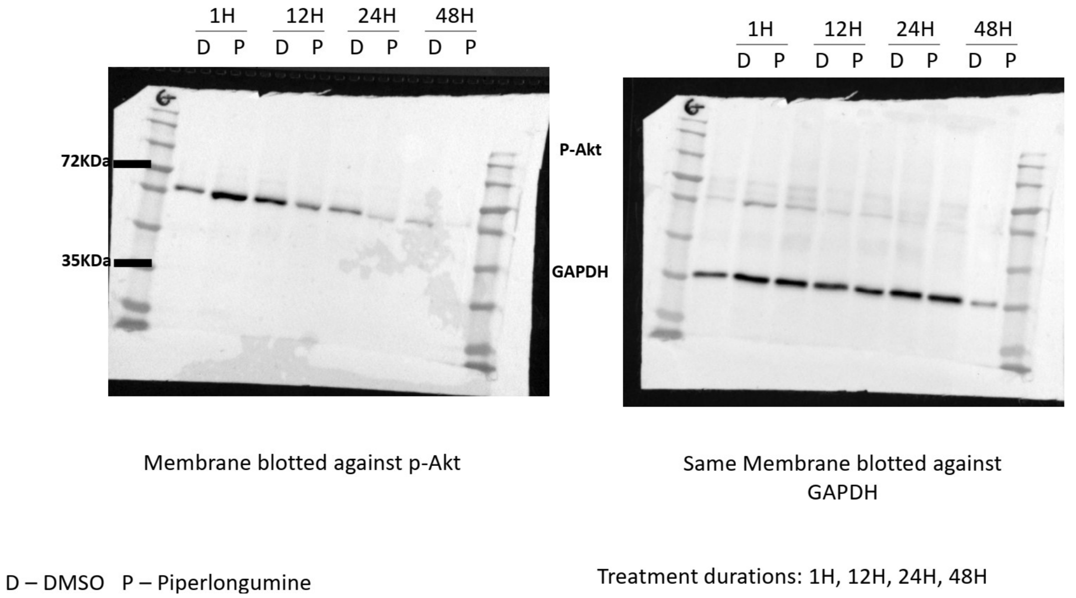

Figure S18. Uncropped Western-Blot for p-Akt and GAPDH in the U2foxRELOC cell line treated with 50 μM PIP (P) for 1, 12, 24 and 48 h. DMSO (**D**) was used as a control for PIP treatment. GAPDH was used as loading control. Refers to Figure 3D.

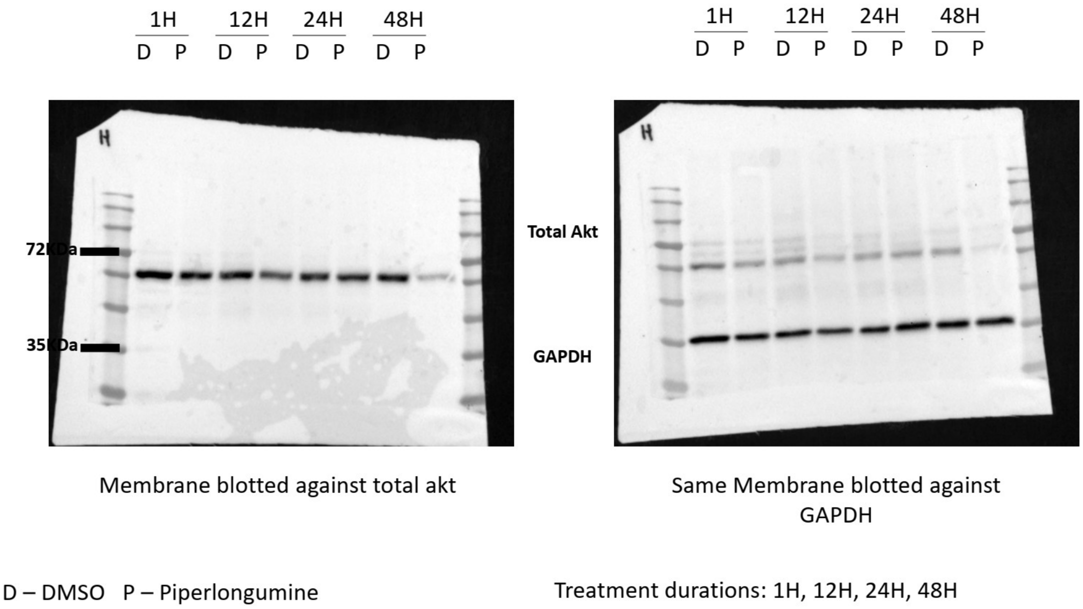

Figure S19. Uncropped Western-Blot for total Akt and GAPDH in the U2foxRELOC cell line treated with 50 µM PIP (P) for 1, 12, 24 and 48 h. DMSO (**D**) was used as a control for PIP treatment. GAPDH was used as loading control. Refers to Figure 3D.

Table S2. Kinase Perturbations Enrichment Analysis grom GEO database (EnrichR tool) TRIB2.

Term	Overlap	*p*-Value	Adjusted P	Old *p*-Value	Old Adjust	Odds Ratio	Combined Score	Genes
PDK1 knockout 80 GSE26290	5/300	0.002364	0.673825	0	0	5.376344086	32.51222507	OLFM1; RASL10A; FAM
GSK3A knockdown 207 GDS4305	4/300	0.014008	1	0	0	4.301075269	18.35759126	CST1; RBPMS; CDH22; K
ITK knockout 241 GSE12465	4/300	0.014008	1	0	0	4.301075269	18.35759126	RBPMS; P2RX1; GNG11

Table S3. Kinase Perturbations Enrichment Analysis grom GEO database (EnrichR tool) BEZ235.

Term	Overlap	*p*-Value	Adjusted P	Old *p*-Value	Old Adjust	Odds Ratio	Combined Score	Genes
PIK3CA mutant 27 GDS4053	2/300	0.029289	1	0	0	7.407407407	26.15209006	PTCH2;ADRA1A
RAGE knockout 269 GSE22873	2/300	0.029289	1	0	0	7.407407407	26.15209006	MYCN;ADRA1A
SYK druginhibition 283 GSE43510	2/300	0.029289	1	0	0	7.407407407	26.15209006	INSIG1;GPD1L
SYK druginhibition 288 GSE43510	2/300	0.029289	1	0	0	7.407407407	26.15209006	FMNL2;INSIG1
EGFR drugactivation 20 GDS2146	2/300	0.029289	1	0	0	7.407407407	26.15209006	ADAMTS15;RGMA
ABL1 knockdown 100 GSE27869	2/300	0.029289	1	0	0	7.407407407	26.15209006	CLMP;FMNL2
CDK19 knockdown 148 GSE32108	2/300	0.029289	1	0	0	7.407407407	26.15209006	MYCN;ADRA1A
ATM knockout 74 GSE23116	2/300	0.029289	1	0	0	7.407407407	26.15209006	CLMP;PDZRN3

© 2020 by the authors. Licensee MDPI, Basel, Switzerland. This article is an open access article distributed under the terms and conditions of the Creative Commons Attribution (CC BY) license (http://creativecommons.org/licenses/by/4.0/).
